# Medical Students and Graduates in the United States during the Session of 1859-60

**Published:** 1860-07

**Authors:** 


### MEDICAL STUDENTS AND GRADUATES IN THE U. S. DURING THE SESSION OF 1859-60.

Below we publish the number of students in attendance at the different medical institutions of this country. It will be seen from this list, which, although it does not represent all our schools, is probably correct, that the classes of the last session were, in the aggregate, much larger than those of the previous one:

| | Students. | Graduates. |
|---|---|---|
| Jefferson Medical College | 630 | 170 |
| University of Pennsylvania | 515 | 173 |
| Pennsylvania Medical College | 130 | 38 |
| University of New York | 411 | 138 |
| College of Physicians and Surgeons | 195 | 55 |
| New York Medical College | 75 | 20 |
| University of Nashville | 401 | 101 |
| Buffalo Medical College | 70 | — |
| Medical Department of Yale College | — | 13 |
| Medical College of Ohio | 123 | 32 |

| | Students. | Graduates. |
|---|---|---|
| Virginia Medical College | — | 82 |
| Medical College of South Carolina | 248 | — |
| Atlanta Medical College | 166 | 56 |
| Savannah Medical College | — | 12 |
| Medical College of Georgia | — | 62 |
| Alabama Medical College | 111 | 15 |
| University of Louisville | 130 | 38 |
| Kentucky School of Medicine | — | 38 |
| Oglethorpe Medical College | 60 | 21 |
| Massachusetts Medical College | 195 | 32 |
| Rush Medical College | 101 | 36 |
| Shelby Medical Colle | 75 | 9 |
| Cleveland Medical College | 70 | 18 |
| Cincinnati College of Medicine and Surgery | 97 | 30 |
| New Orleans School of Medicine | 216 | 63 |
| St. Louis Medical College | — | 52 |
| Missouri Medical College | — | 30 |
| University of Maryland | — | 50 |
| University of Louisiana | 401 | 113 |
| University of Michigan | 185 | — |
| University of Virginia | 104 | — |

